# NVivo

**DOI:** 10.5195/jmla.2022.1271

**Published:** 2022-04-01

**Authors:** Kerry Dhakal

**Affiliations:** 1 kerry.dhakal@osumc.edu, Assistant Professor, Research and Education Librarian, Health Sciences Library, The Ohio State University, Columbus, OH

## Abstract

**NVivo.** A qualitative data analysis software tool, QSR International, https://www.qsrinternational.com/nvivo-qualitative-data-analysis-software/home; $1,249/user (one-time cost for using specific version indefinitely; upgrades are additional).

## INTRODUCTION

Qualitative data can produce meaningful findings if they are managed properly, even though these data can oftentimes be multifaceted [1]. Given the different types of qualitative data, tools to manage and analyze this information must provide options for data in multiple formats. NVivo can import and support multiple types of formats and data types and is a helpful tool for sorting, organizing, and analyzing qualitative data. It has also been argued that using NVivo or computer-assisted qualitative data analysis software (CAQDAS) can even improve the quality of the analysis [3].

NVivo is a CAQDAS program. CAQDAS programs assist qualitative researchers to collect, organize, analyze, visualize, and report their data. CAQDAS programs do not, however, replace the need for the human researcher; they assist the researcher by offering tools and features to organize and structure the data collected. Other CAQDAS programs include Atlas.ti (Scientific Software Development GmbH), Dedoose (SocioCultural Research Consultants/UCLA), and QDA Miner (Provalis Research), among several others [2]. The audience for all CAQDAS programs, including NVivo, is for qualitative researchers, mixed-methods researchers, and students learning about qualitative and mixed-methods research data collection, analysis, display, and reporting.

## NVIVO FUNCTIONALITY

NVivo software is a program created by QSR International. The first version of NVivo (1.0) was released in 1997. Currently, the most recent version of NVivo available is simply called “NVivo” and was released in March 2020. The version being reviewed here is NVivo 12 Pro, which was created in 2018 and is still being used and supported by QSR International. The system requirements for using NVivo 12 Pro on Microsoft Windows includes using a version of Windows 8.1 or higher with 64-bit support. In an Apple Mac operating system (macOS), NVivo can work only in macOS 10.13 or higher. For individual licenses, QSR allows users to download NVivo software on two separate devices. If researchers are going to import data into NVivo from other data analysis software programs, such as SPSS or Microsoft Excel, QSR International suggests having those programs downloaded on the same device where NVivo is downloaded.

## FACILITATING DATA MANAGEMENT AND ANALYSIS

NVivo's import tool can bring in several document file types, including PDFs, Microsoft Office files, and statistical and textual data files. It can also import digital and scanned images and videos. The user can also import another NVivo project and its files or use the NCapture tool to import social media data. Since qualitative researchers often do end up analyzing a variety of data types in their analysis process, being able to organize and temporarily store such data and document types in NVivo makes for easier preparation of data for analysis, display, and reporting.

## CODING AND CATEGORIZATION TOOLS

The first phase of analysis in qualitative research is coding of the datasets. Coding essentially means labeling and creating categories for sections or “chunks” of data in the dataset. For librarians, this process would be similar to creating subject headings labels for sections of data that may help move forward or bring to the surface emerging themes in the second phase of analysis. One of the benefits of using NVivo 12 Pro is that it has developed several options for users to sort, label, and organize coded data hierarchically through the node and through classification and mapping tools. Nodes are the name that NVivo uses for codes, and there are two types of nodes: thematic nodes and case nodes. Classification tools allow users to create categories of data that are from one source or multiple cases that have attributes and values data and allow the user to map thematic data to case data. Mapping tools include templates and visual representations for users to interact with and fill in with data and relationships established between chunks of data. These coding, classification, and mapping tools promote additional organization to the data so that the researcher can query the data to analyze it, draw conclusions, and verify findings across all units of analysis.

Thematic coding in NVivo is easily performed by selecting a section of text from a source document, like an interview transcript or from an image or other type of source document, and then tagging it with a node. The node can either be created in situ or ahead of time. NVivo users can create as many nodes as they feel are necessary and even double code a section of text or image as they see fit. Color-coding is also available in NVivo for codes. That is, users can assign colors to specific codes and then can view the colors coded to identify gaps or themes with the colors. The only limiting aspect of this tool is that there are not many colors to choose from, so if many colors are needed, the user needs to be selective in when to use them. In the nodes tool, researchers can develop parent codes and child codes. One can also create a folder structure in the nodes tool to have a folder for initial coding and another for final coding to organize the coding process in a meaningful way.

Other tools to categorize and start analyzing the data in NVivo include Sentiments and Relationship Types. Sentiments allow the user to tag data that they consider positive or negative, in a way that is meaningful to the user or researcher. The Relationship Types tool allows users to indicate if the relationship between two points or sections of data are associated in different ways. Users can specify that the relationship is associative in general or that is it unidirectional or bidirectional. The ability to color-code, apply relationship and sentiments, and organize meaningful data by codes, cases, and classification really illustrates why NVivo is helpful. It allows the user to see their data both at a global view and in a very detailed view that may not be accessible easily to researchers not using NVivo.

## CLASSIFICATIONS

The file classification tool in NVivo 12 Pro essentially allows the user to add metadata in a structured way for coded data. One way of preparing and conducting analysis is coding data files, sections of files or text, or other data like images or videos with nodes. Another way to organize the data and files is to create file classifications that allow the user to develop broader categories of files that can be organized by metadata or categorical attributes for further analysis. For example, a file classification might be Interviews, and all the interview transcripts as a file document would be classified as such before coding starts. A case classification would be used to organize the interviews from a specific group or organization (e.g., nursing students at Hudson University). For example, the user may have four cases that are interview transcripts from a group of nurses. Each interview document is named/coded by the interviewee's name as a case and then the classification may be named for the university the students attend.

Another way to code in NVivo is by case coding. Case coding can be found in the nodes tool. Mainly, case codes are used to capture demographic information about people, places, or units of analysis that have attributes. For example, if interviews are with two people, Monique and John, the user could code those interviewees as “People” (Table 1). The user could then code (or provide) attributes that might need to be further analyzed about those people in the case coding tool. The case node tool then creates a spreadsheet to accommodate the attributes as rows and provides cells next to it for the user to input the values of those attributes.

**Table 1:** Case node examples

**Case Node: People (Monique)**	
Attributes	Values
Age	26
Gender	Female
Major	Nursing
**Case Node: People (John)**	
Attributes	Values
Age	22
Gender	Male
Major	Optometry

Ultimately, classifications offer the chance to add broader categories of organization and hierarchical depth to the data analysis process.

## VISUALIZATIONS, QUERIES, AND REPORTING

The approach of Miles and Huberman to qualitative data analysis follows three broad steps including data reduction, data display, and drawing conclusions and verifying them [1]. Data reduction includes what we have discussed so far concerning the preparation of raw data to be analyzed using nodes (codes), cases, classifications, and linking cases and coded data to learn what themes emerge from the analysis of the data. Additionally, NVivo has several features in its cadre of tools that ease the process of finding relationships between chunks of data as themes begin to emerge. These tools include the ability to query the data to search for codes or for word usage, develop a framework matrix, and visualizing relationships between coded data through mind maps, concept maps, and project maps.

## NVIVO AND LIBRARIANS

Academic librarians from around the globe are taking the initiative to learn CAQDAS, including NVivo, to enhance the services they offer to researchers and students [3, 4, 5]. Several libraries now have LibGuides providing information on how to use NVivo for data analysis. Some provide links to webinars offered by QSR International or teach courses themselves in collaboration with qualitative researchers or research educators [3, 4] and others offer consultations. Additionally, librarians are themselves conducting research and using NVivo software to analyze their qualitative or mixed-methods data [6–9]. These examples demonstrate that librarians can enhance their research skill set, offer training, and continue to develop their role as important collaborators in the research lifecycle and in interprofessional education. This finding supports research discussing the skills needed by librarians to support research faculty [10–13]. It also supports the indicators for developing expertise for Competency 5: Evidence-Based Practice and Research, from the Medical Library Association's Competencies for Lifelong Learning and Professional Success [13].

## COST

QSR International offers a free trial of the NVivo software. The individual license for the software is a one-time cost of $1,249 to use a particular version of the software indefinitely. There are, however, additional costs for upgrades. Institutional licenses vary depending on the number of users. Annual subscriptions offer the best value and include the cost for any upgrades.

## TRAINING AND SUPPORT

QSR International provides several options for training and support. On the homepage of NVivo 12 Pro is the Learn and Connect resources. These resources link users to tutorials and contact information to ask troubleshooting questions. QSR also has an NVivo Research Network that licensed users can join to participate in occasional webinars for no extra cost and a certificate program where licensed users can pay for additional training that is more in-depth [14].

## CONCLUSION

NVivo is a computer software program that allows researchers to manage, analyze, and visualize qualitative data and documents systematically and individually. It is user-friendly for researchers who are familiar with coding and qualitative data analysis strategies. There is a little upfront learning about coding and thematic analysis options for beginner researchers. However, QSR International has helpful resources and support for those who need it, as well as colleagues who are using and teaching it. This reviewer recommends NVivo 12 Pro as one of the qualitative data software programs to use for experienced qualitative researchers and as a learning tool for students of qualitative research methodologies.
